# Determining the Affinity and Kinetics of Small Molecule Inhibitors of Galectin-1 Using Surface Plasmon Resonance

**DOI:** 10.3390/ijms25126704

**Published:** 2024-06-18

**Authors:** Henry Kim, Louis Kretz, Céline Ronin, Christina Starck, James A. Roper, Barbro Kahl-Knutson, Kristoffer Peterson, Hakon Leffler, Ulf J. Nilsson, Anders Pedersen, Fredrik R. Zetterberg, Robert J. Slack

**Affiliations:** 1 NovAliX, 16 Rue d’Ankara, 67000 Strasbourg, France; 2 Galecto Biotech AB, Stevenage Bioscience Catalyst, Stevenage SG1 2FX, UK; 3 Department of Laboratory Medicine, Lund University, P.O. Box 124, SE-221 00 Lund, Sweden; 4 Galecto Biotech AB, Sahlgrenska Science Park, Medicinaregatan 8 A, SE-413 46 Gothenburg, Sweden; 5 Department of Chemistry, Lund University, P.O. Box 124, SE-221 00 Lund, Sweden; 6 Galecto Biotech AB, Cobis Science Park, Ole Maaloes Vej 3, DK-2200 Copenhagen, Denmark

**Keywords:** galectin-1, small molecule glycomimetics, surface plasmon resonance, fluorescence polarization

## Abstract

The beta-galactoside-binding mammalian lectin galectin-1 can bind, via its carbohydrate recognition domain (CRD), to various cell surface glycoproteins and has been implicated in a range of cancers. As a consequence of binding to sugar residues on cell surface receptors, it has been shown to have a pleiotropic effect across many cell types and mechanisms, resulting in immune system modulation and cancer progression. As a result, it has started to become a therapeutic target for both small and large molecules. In previous studies, we used fluorescence polarization (FP) assays to determine *K_D_* values to screen and triage small molecule glycomimetics that bind to the galectin-1 CRD. In this study, surface plasmon resonance (SPR) was used to compare human and mouse galectin-1 affinity measures with FP, as SPR has not been applied for compound screening against this galectin. Binding affinities for a selection of mono- and di-saccharides covering a 1000-fold range correlated well between FP and SPR assay formats for both human and mouse galectin-1. It was shown that slower dissociation drove the increased affinity at human galectin-1, whilst faster association was responsible for the effects in mouse galectin-1. This study demonstrates that SPR is a sound alternative to FP for early drug discovery screening and determining affinity estimates. Consequently, it also allows association and dissociation constants to be measured in a high-throughput manner for small molecule galectin-1 inhibitors.

## 1. Introduction

The galectin family is made up of 15 proteins and contains the beta-galactoside-binding mammalian lectin galectin-1 that binds cell surface glycoproteins via its carbohydrate recognition domain (CRD), resulting in the modulation of cell–cell and cell matrix interactions [1]. Galectin-1 has been shown to be a key modulator of the immune system and, in the progression of cancer, has resulted in it being a focus for therapeutic targeting [2,3]. In the context of cancer, the interaction of galectin-1 with glycosylated tumour-associated receptors, e.g., growth factor receptors and integrins, has been shown to control tumour cell fitness, migration, stemness and epithelial-to-mesenchymal transition in addition to angiogenic effects on endothelial cells and apoptosis of immunomodulatory T cells [4]. In addition, increased expression and an association with survival have been demonstrated for galectin-1 across a range of cancer types [5]. 

The targeting of galectin-1 from a drug discovery perspective, as is the case for the majority of the galectin family, with the exception of galectin-3 [6] and -9 [4], has, to date, been limited. However, there has been some traction with the beginnings of the identification of selective small molecule inhibitors and therapeutic monoclonal antibodies [7,8], although none have yet made it to clinical testing. 

In previous studies, we used fluorescence polarization (FP) to assess small molecule glycomimetics binding galectin-1 and measuring affinity (*K_D_*) and structure–activity relationships (SAR) [9]. As detailed in our recent study, where as part of a small molecule lead optimization project to find galectin-3 inhibitors, the comparison of SPR with FP was investigated [10], in this study, we endeavoured to explore the same comparisons between assay formats and across species for galectin-1. The aims were therefore similar where we compared our primary FP SAR assay for the project with an SPR counterpart to compare the binding affinities and kinetics of novel glycomimetics between human and mouse protein as well as validate for the measurement of *k_on_* and *k_off_* for this galectin in a high-throughput manner. This would allow a set of assays to be available to enable SAR properties for small molecule galectin-1 CRD binders to be compared, not only for affinity but also for association and dissociation kinetics across a much lower concentration range than previously demonstrated, in addition to investigating cross-species differences. Consequently, this would also enable further insight into our novel galectin-1 small molecule drug discovery efforts to develop more optimal mouse and human inhibitors for preclinical and clinical investigation. 

## 2. Results

**Figure 1:**
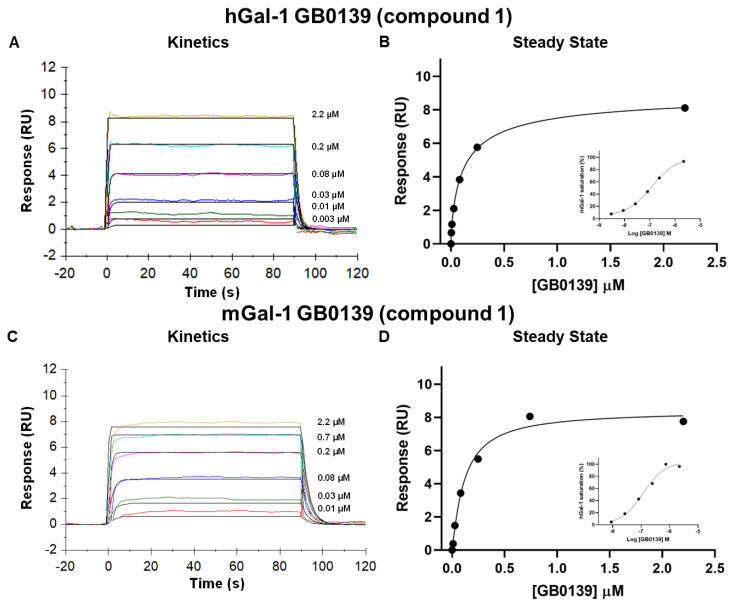
GB0139 sensorgrams demonstrating human (**A**) and mouse (**C**) galectin-1 binding kinetics in SPR. The global fit of the 1:1 Langmuir interaction model is shown in solid black lines, with raw data shown in the coloured lines. Binding at steady states is also displayed on a linear and logarithmic scale (inset) (**B**,**D**) with % galectin saturation normalized to baseline and the highest saturating concentration of GB0139. hGal-1, human galectin-1; mGal-1, mouse galectin-1.

**Figure 2:**
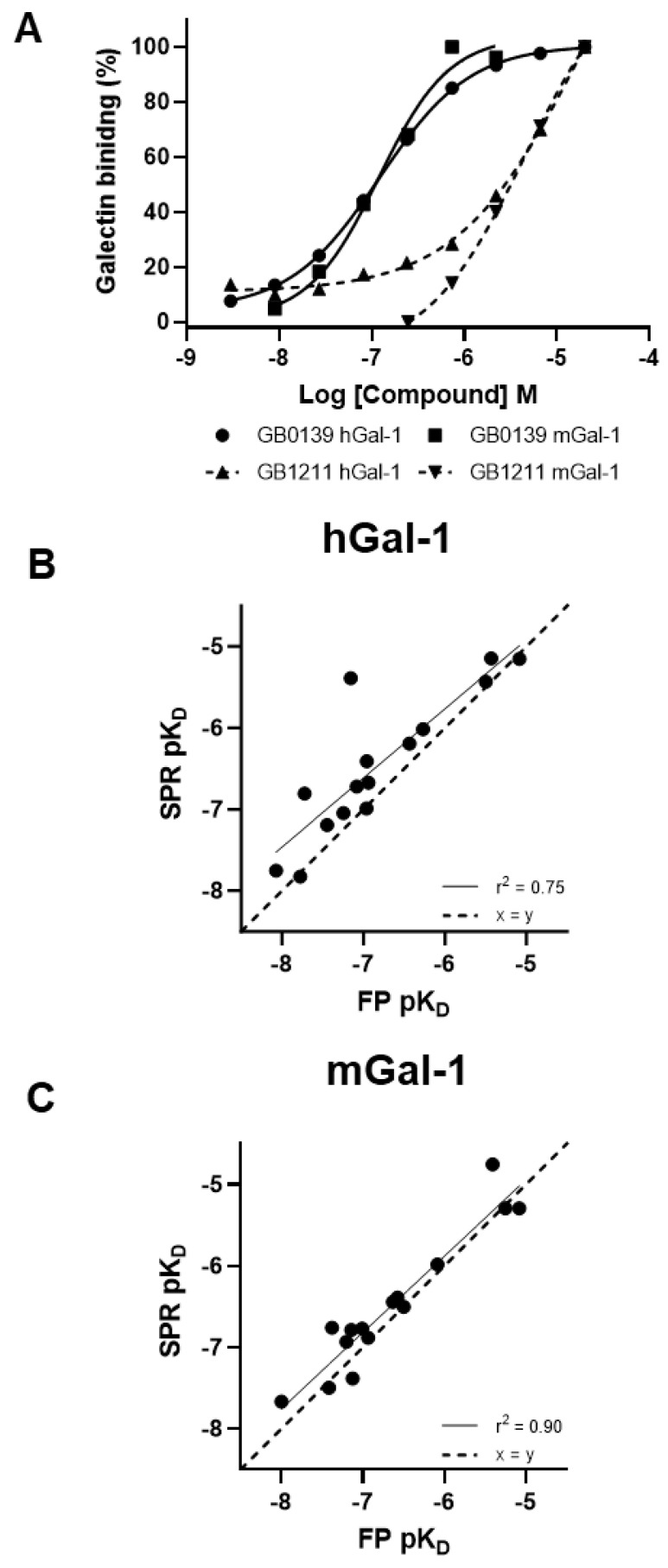
GB0139 and GB1211 steady-state binding to galectin-1 (human and mouse) measured in SPR (**A**) with the highest concentration (saturation) of compound and baseline levels used to normalize % galectin binding. Glycomimetic small molecule affinity correlations from SPR (steady-state analysis) and FP are shown vs. human (**B**) and mouse (**C**) galectin-1. hGal-1, human galectin-1; mGal-1, mouse galectin-1; *pK_D_*, log_10_ of *K_D_*; ss, steady state.

**Figure 3:**
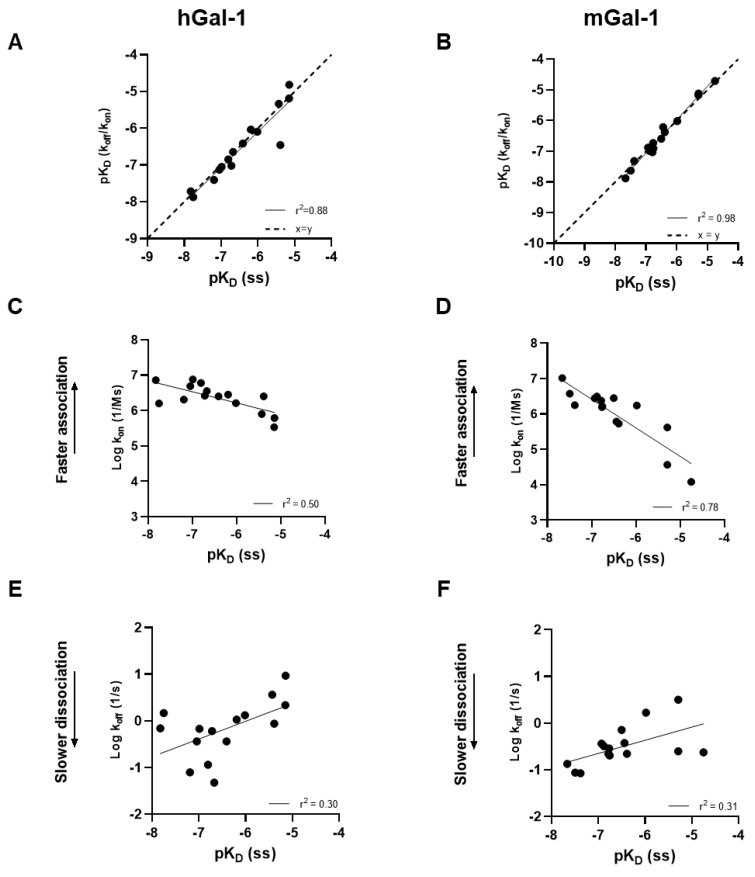
Glycomimetic small molecule affinity (*pK_D_*) correlations determined via kinetic and steady-state analysis against human (**A**) and mouse (**B**) galectin-1 in SPR. Association or dissociation rate (log_10_) correlation with human (**C**,**E**) and mouse (**D**,**F**) galectin-1 affinity also shown. *pK_D_*, log_10_ of *K_D_*; ss, steady state.

**Figure 4:**
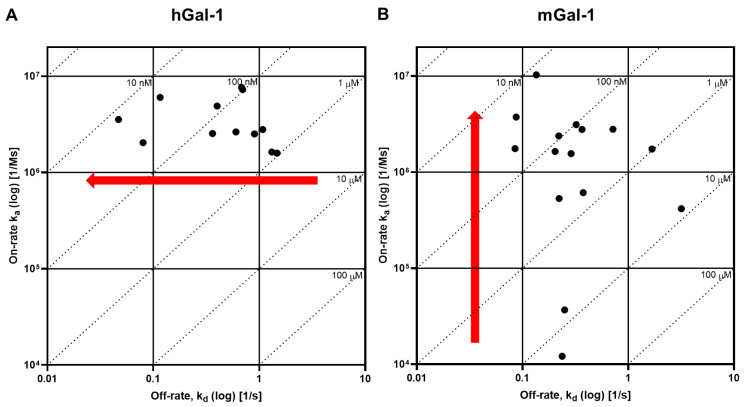
Association and dissociation rate (log_10_) correlations for human (**A**) and mouse (**B**) galectin-1. On the top and right-hand side of each panel, on the intersection of association and dissociation rates, *K_D_* values are shown (calculated by *k_off_*/*k_on_*). Red arrows show direction of trends for kinetic parameter that drives affinity change in each species.

**Figure 5:**
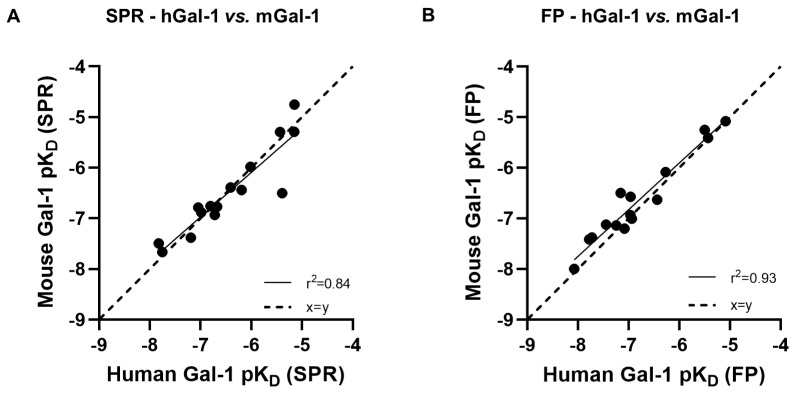
Glycomimetic affinity correlations for human and mouse galectin-1 measured in either SPR (**A**) or FP (**B**). *pK_D_*, log_10_ of *K_D_*.

A galectin-1 human and mouse protein SPR assay was used to screen 15 glycomimetic small molecules (see Supplementary Materials). These consisted of mono- and di-saccharides with a 965-fold range determined in FP binding assays. Overall, a higher affinity was observed for disaccharides against galectin-1 human and mouse protein in both the SPR and FP assays. A single phase of binding upon the association to and dissociation from galectin-1 in SPR was shown for all compounds (sensorgram data for all compounds tested in all SPR assays can be found in Supplementary Materials), with examples shown for GB0139 (compound **1**, disaccharide) [11] (Figure 1 and Figure 2A) and GB1211 (compound **3**, monosaccharide) [12] (Figure 2A). For human and mouse galectin-1, the GB0139 kinetic *K_D_* values in SPR were determined to be 103 nM and 131 nM, respectively (Figure 1 and Figure 2). The kinetic and steady-state SPR K_D_ values for both human (r^2^ = 0.88) and mouse (r^2^ = 0.98) assays were closely correlated (Figure 3A,B). Between FP and SPR assays, the compound rank order was well correlated between human and mouse galectin-1 assays with r^2^ values of 0.75 and 0.90, respectively (Figure 2B,C). In the human galectin-1, a mean *K_D_* fold shift of 5.9 to lower sensitivity in the SPR assay was demonstrated vs. the FP assay, whilst this was a more comparable set of data for the mouse galectin-1 between the two assay formats (mean fold shift of 1.7 from SPR to FP). A decrease in (or slower) dissociation rate was correlated with the affinity increase for glycomimetic small molecule binding to human galectin-1 (Figure 3E). Conversely, an increase in (or faster) association rate but no effect on dissociation rate was correlated with an affinity increase for a glycomimetic small molecule to mouse galectin-1 (Figure 3D,F). The increase observed in association with the human galectin-1 (1 log unit) was much less than that seen for mouse galectin-1 (3 log unit) (Figure 3C,D). The correlation of *k_on_* and *k_off_* values for each galectin-1 species (Figure 4A,B) further highlighted these observations. When comparing glycomimetic affinities within each assay format from human to mouse galectin-1, a good correlation was observed (Figure 5A,B). When comparing species within each assay format, the correlations were also very comparable (SPR assay format r^2^ = 0.84; FP assay format r^2^ = 0.93).

## 3. Discussion

The pleiotropic nature of galectin-1 in a number of cancers was demonstrated, with lectin inducing detrimental effects across several key mechanisms and cell types [4,5]. As a result, it is starting to become a focus for drug targeting in cancer. Even though the weight of scientific evidence is building, no galectin-1 inhibitor has been fully developed for testing in humans. However, recently, small and large molecule galectin-1 inhibitors have been discovered and have now started to be investigated in pre-clinical studies [7,8].

Affinity measurements of galectin-1 small molecule glycomimetics in our studies have been historically completed using FP methods. The application of SPR to galectin-1 has been primarily focused on the investigation of large molecule and protein–protein interactions [13,14,15]. There are examples of low-affinity small molecule interactions with galectin-1 measured using SPR, although this is limited to individual compounds with limited information on assay methods [16]. A more in-depth investigation and comparison of the measurement of small molecule interactions across a broader affinity range (µM to nM) has been limited to galectin-3 and our historical study [10]. Therefore, in this study, we applied the same approach to galectin-1, whereby, as a substitute, we utilized SPR binding assays to provide confirmation to FP as well as obtain a high-throughput measurement of binding kinetics for a set of galectin-1 inhibitors and allow comparisons between species (human vs. mouse).

As would be expected, when a compound array of 15 compounds made up of mono- and di-saccharides exemplars were tested in SPR assays to determine steady-state and kinetic-derived *K_D_* values, an excellent correlation was seen for human and mouse galectin-1 systems. Subsequently, when comparing affinity values between SPR and FP, steady-state *K_D_* estimates were used, and for both species, the SPR values showed a close agreement with those determined in FP.

Although there was no difference in the affinities of compounds for the CRD site on the human and the mouse forms of galectin-1, a difference in the kinetics between the species was observed. For human galectin-1, a decrease in dissociation rate for small molecule glycomimetics was correlated with an increase in affinity. In contrast, affinity increases in the binding to mouse galectin-1 were not a result of a change in dissociation rate but rather an elevation in association rate. In addition, the increase was much more marked for the association rate with the mouse galectin-1 compared to the dissociation rate change in the human form. This is an interesting and novel observation, albeit in a small compound set, and it is not immediately clear what drives this in the set of glycomimetics investigated in this study. Although out of the scope of this study, it would be interesting to further investigate this phenomenon with a larger set of glycomimetic small and large molecules in the SPR assay to understand the SAR associated, as well as bring in other techniques, e.g., X-ray crystallography and computational docking, to elucidate what properties drive this observation.

In summary, for early drug discovery initiatives focused on the identification of small molecule CRD binders of galectin-1, SPR as a screening approach has been validated and shown to be a solid replacement for, or a confirmation of, affinity measures in FP assays. The added benefit of the use of SPR is that high-throughput and *k_on_* and *k_off_* values can be generated for much earlier kinetic characterization of galectin-1 small molecule glycomimetics and inhibitors. The novel compound set investigated in this study covers a range of affinities from µM to nM that have not been investigated before using SPR. This has resulted in data showing the comparison of association and dissociation kinetics across this wider concentration range as well as cross-species between mice and humans. This is the first time this has been completed for this target and has identified differences in what drives affinity between species. Historically, it has been a challenge for other galectins with differences between humans and rodents, e.g., galectin-3 [10]. Therefore, we believe this work is of interest to other groups working in this space in academia and industry to allow further focus on the SAR that drives these differences and enable improved therapeutic inhibitors to be developed in the future in the preclinical and clinical space.

## 4. Materials and Methods

### 4.1. Materials

**Figure 6:**
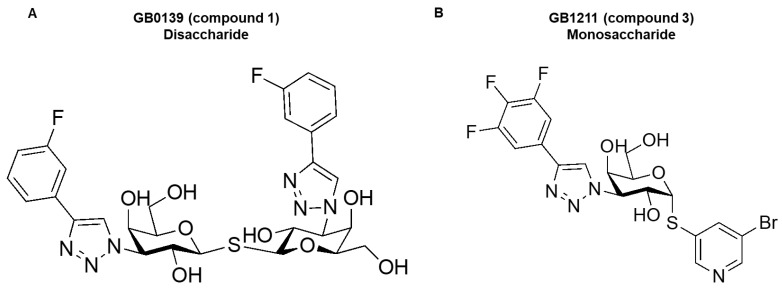
The disaccharide (GB0139 compound **1**) (**A**) and monosaccharide (GB1211 compound **3**) (**B**) chemical structures as examples from the compound sets tested in this study.

In this investigation, glycomimetic small molecules had molecular weights ranging from 481 to 676 Da and were synthesized by the Medicinal Chemistry Department at Galecto Biotech AB (Gothenburg, Sweden). Compound screening arrays were made up of disaccharides and monosaccharides (see Supplementary Tables S1 and S2), with exemplars highlighted for each series in Figure 6 (disaccharide GB0139 [11] in Figure 6A and monosaccharide GB1211 [12] in Figure 6B) for comparison across 2 chemical series under SAR investigation. All compound stocks were made up of 100% DMSO at 10 mM. The maximum concentration tested in the SPR assay (described below) was chosen based on compound solubility or critical aggregation concentration (CAC), where available. All SPR reagents were purchased from Cytiva (Marlborough, MA, USA). All other reagents were purchased from Sigma-Aldrich (St. Louis, Missouri, United States) unless otherwise stated.

### 4.2. Protein Generation

For SPR studies, His-tagged proteins were expressed in *E. coli* strain BL21 (human galectin-1 (residues M1-D135) and mouse galectin-1 (residues M1-E135)) and purified as previously described [10]. SDS-PAGE and mass spectrometry were used to confirm protein purity with all proteins stored at −80 °C until use. Human and mouse proteins for FP were produced as previously described [9].

### 4.3. Surface Plasmon Resonance (SPR) Binding Assays

The SPR method previously described for galectin-3 proteins was used here for galectin-1 [10]. Briefly, CM3 sensor chip surfaces (Cytiva, Marlborough, MA, USA) were pre-primed and equilibrated in immobilization buffer (10 mM HEPES, 150 mM NaCl (pH 7.4)) using an Amine Coupling Kit (Cytiva, Marlborough, MA, USA) prior to the immobilization of protein (injected at 10 µg/mL in PBS at pH 6.0). Chip surfaces were activated with 1-ethyl-3-[3-dimethylaminopropyl]carbodiimide hydrochloride (EDC)/N-hydroxysuccinimide (NHS) at a flow rate of 5 µL/min for 420 s prior to protein injections (10 µL/min flowrate) to generate 400–800 response units (RU). Chips surfaces were blocked with ethanolamine HCl (pH 8.5) for 420 s upon reaching the immobilization required. Then, EDC/NHS and ethanolamine were used to treat blank surfaces that were protein-free. Running buffer (RB) was used to dilute compounds (10 mM HEPES, 150 mM NaCl, 1 mM DTT, 0.05% Tween 20, 5% DMSO (pH 7.4)), pre-filtered, degassed, and then injected for 1 min at a flow rate of 30 μL/min. Minimal non-specific binding was observed between compounds and the blank reference surfaces. Following each cycle, the chip surface was auto-regenerated and aided by fast compound off-rates, and a further wash set was completed in RB. After 3 min of dissociation, the baseline returned to the starting level, and during runs, there was no evidence of surface accumulation or signal loss. H_2_O/50% DMSO was used to wash the flow system following each cycle, and there were no signs of cross-contamination in the carryover control. At the start and the end of each run, solvent correction was completed and included in the data analysis. There were no observed mass transport limitation effects. To characterize binding between compounds and galectin-1 protein (all carried out at 25 °C), Multi Cycle Kinetics (MCK) and steady-state affinity analyses were completed. A Biacore™ T200 (Cytiva, Marlborough, MA, USA) was used for all SPR experiments. 

### 4.4. Fluorescence Polarization Binding Assays

FP assays for measuring CRD binding to galectin-1 were completed as previously described [9,17]. Briefly, the FP signal was detected in black polystyrene 96-well microtiter plates (Corning, Glendale, AZ, USA) using a PolarStar instrument (BMG, Offenburg, Germany) with a probe and galectin (100 μL/well) at fixed concentrations (detailed in [8,9]) incubated with inhibitor solution (100 μL/well). For controls, fluorescein or fluorescent probe wells were included. For reagent dilution, PBS was used, and plates were incubated for 1 h prior at room temperature for the measurement of FP. 

### 4.5. Data Analysis

To analyze SPR MCK and affinity data, Biacore™ T200 Evaluation Software (Cytiva, Marlborough, MA, USA) was used by applying a simple interaction model (1:1 Langmuir) to determine affinities (*K_D_*) and kinetics (data globally fitted to determine association (*k_on_*)/dissociation (*k_off_*) rate constants) of the compound–protein. *k_off_* was divided by *k_on_* to determine kinetically derived SPR *K_D_* values. To account for background buffer and bulk-shift effects, reference flow cell signals were subtracted from each data set. In FP assays, *K_D_* values for inhibitor–galectin interactions were determined by solving the two mass action equations describing galectin–inhibitor and galectin–probe interactions directly from single data points as previously described [9]. The largest *K_D_* was divided by the smallest to calculate fold differences in affinity between assays. Data visualization was completed in GraphPad Prism version 10 (GraphPad Software, Boston, MA, USA).

## Data Availability

The data presented in this study are available on request from the corresponding author.

## Supplementary Materials

The following supporting information can be downloaded at: https://www.mdpi.com/article/10.3390/ijms25126704/s1 [18,19].
