# Supplementary material for: Determining the Affinity and Kinetics of Small Molecule Inhibitors of Galectin-1 Using Surface Plasmon Resonance
Source: Int J Mol Sci. 2024 Jun 18;25(12):6704. doi: 10.3390/ijms25126704 (PMC11203799; doi:10.3390/ijms25126704)
Supplement: Supplementary file 1 [file ijms-25-06704-s001.zip › ijms-2989282-supplementary.pdf]

Supplementary Figures

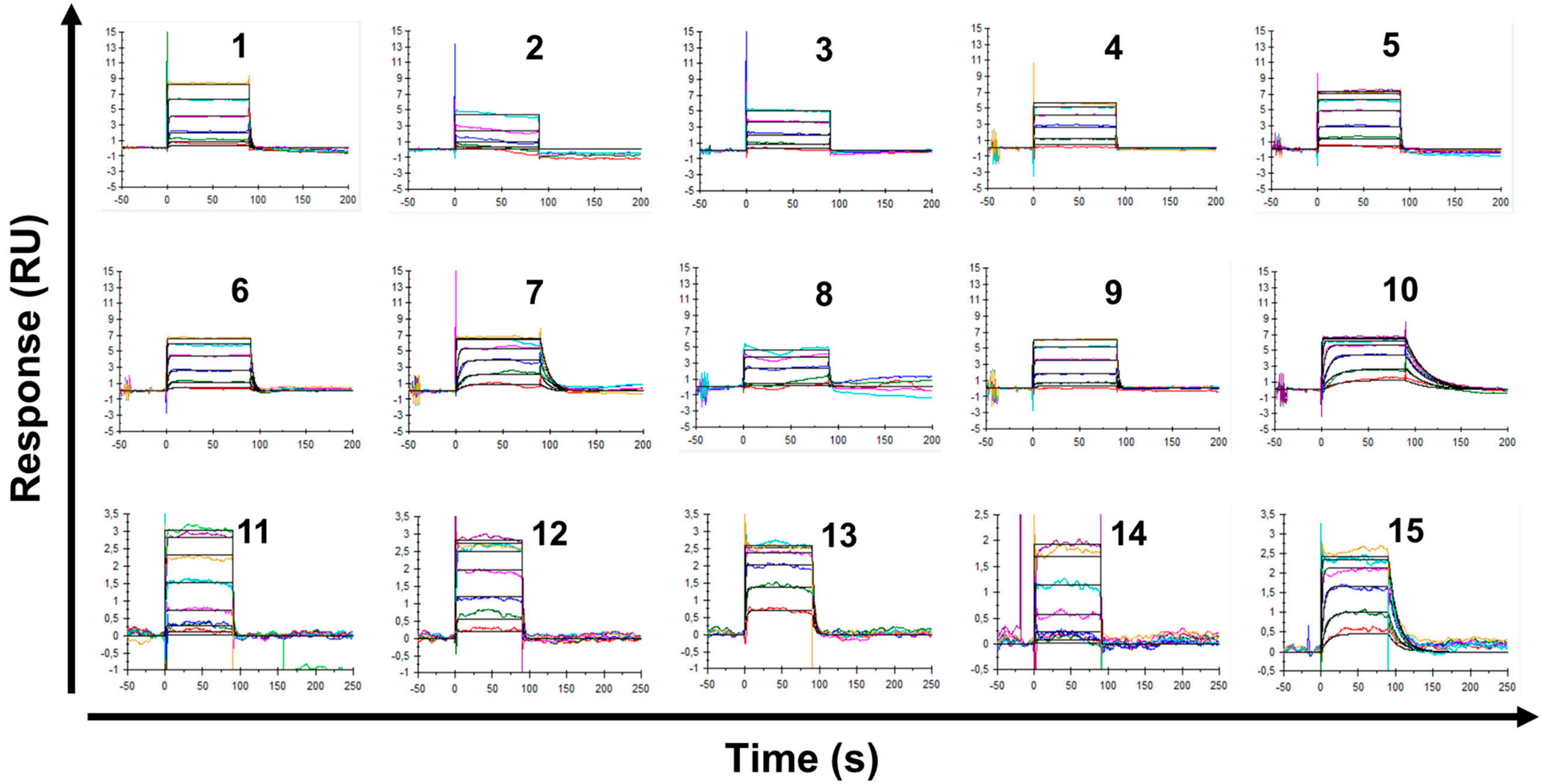

Supplementary Figure S1. SPR sensorgrams showing kinetics of all compounds binding to human galectin-1. Coloured lines show the raw data whilst solid black lines shows global fit of 1:1 Langmuir interaction model (see Table S1 for compound references). Compound concentration ranges tested (1:3 dilutions) were between 0.003 and 40 μM.

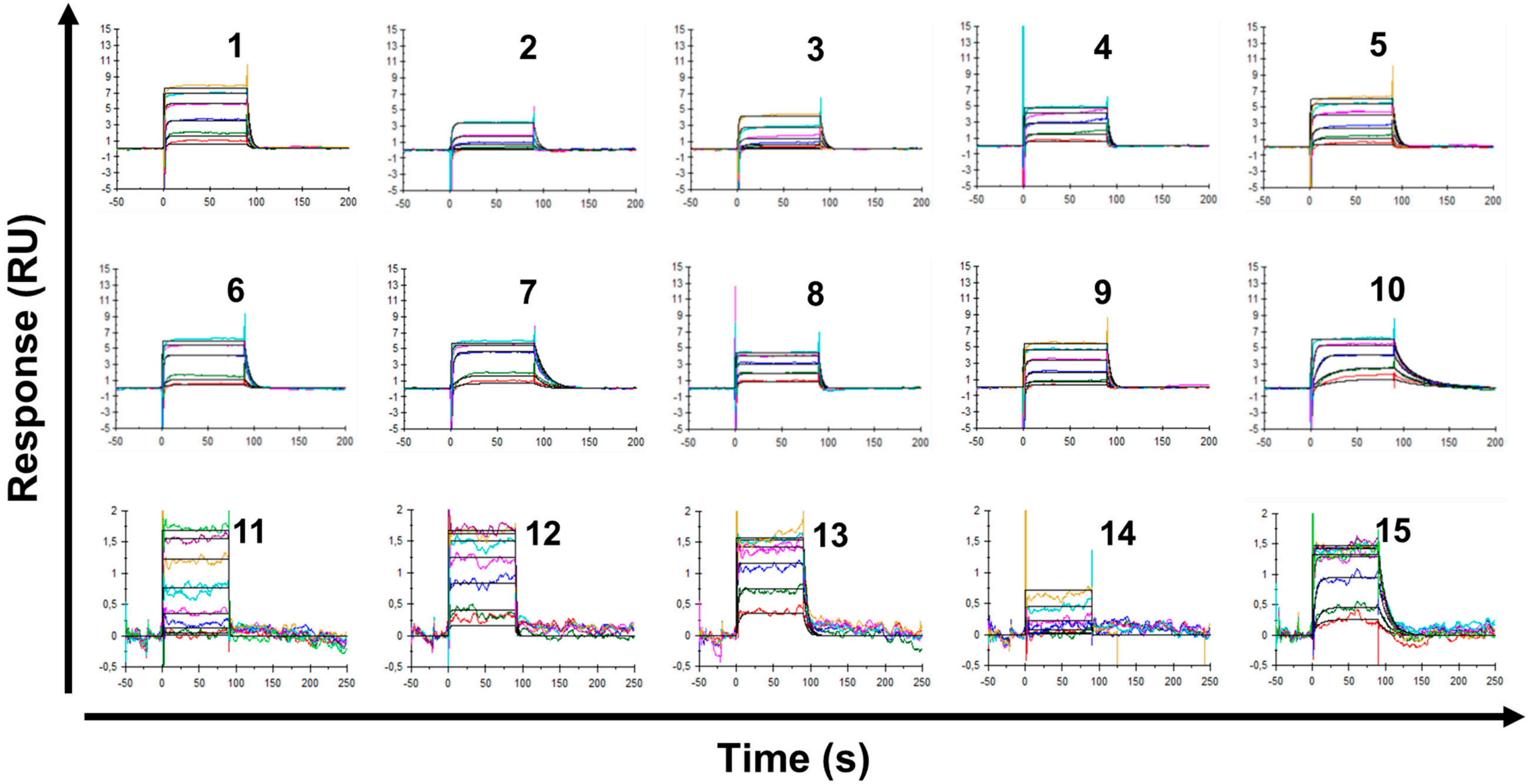

Supplementary Figure S2. SPR sensorgrams showing kinetics of all compounds binding to mouse galectin-1. Coloured lines show the raw data whilst solid black lines shows global fit of 1:1 Langmuir interaction model (see Table S2 for compound references). Compound concentration ranges tested (1:3 dilutions) were between 0.003 and 20 μM.

Supplementary Results

Supplementary Table S1. Binding parameters determined for all compounds against human galectin-1 in FP and SPR assays.

Compound ID	Structure	Compound Series	FP Mean K_D (μM) ± SD[+] (n)	SPR ss K_D (μM)	SPR kinetic K_D (μM)	SPR *kon* (x10^6 1/Ms)	SPR *koff* (1/s)
**1* (GB0139 [11])**	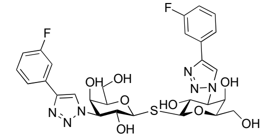	Disaccharide	0.109 ± 0.022 (>3)	0.103	0.089	7.65	0.681
**2* (GB1107 [18])**	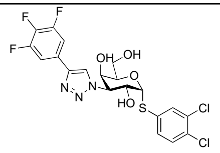	Monosaccharide	3.70 ± 0.15 (>3)	7.14	15.2	0.612	9.30
**3* (GB1211 [12])**	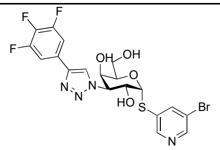	Monosaccharide	3.17 ± 0.16 (>3)	3.69	4.62	0.786	3.63
**4* (GB1490 [19])**	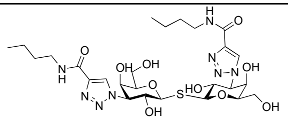	Monosaccharide	0.366 ± 0.177 (3)	0.644	0.925	1.59	1.47
**5**	NA	Disaccharide	0.011 (1)	0.392	0.386	2.80	1.08
**6**	NA	Monosaccharide	0.019 (1)	0.157	0.143	2.54	0.362

**7**	NA	Monosaccharide	0.017 ± 0.008 (>3)	0.015	0.019	6.02	0.116
**8**	NA	Monosaccharide	0.083 (1)	0.192	0.096	7.26	0.697
**9**	NA	Monosaccharide	0.116 (0.087, 0.144)	0.213	0.228	2.64	0.602
**10**	NA	Monosaccharide	0.009 (0.008, 0.009)	0.018	0.013	3.55	0.047
**11**	NA	Disaccharide	0.54 ± 0.17 (3)	0.968	0.807	1.63	1.31
**12**	NA	Disaccharide	0.07 (1)	4.07	0.349	2.51	0.877
**13**	NA	Monosaccharide	0.057 ± 0.010 (>3)	0.090	0.075	4.90	0.366
**14**	NA	Disaccharide	8.20 (1)	7.07	6.46	0.335	2.17
**15**	NA	Monosaccharide	0.036 (0.034, 0.038)	0.064	0.039	2.04	0.080

*Literature name (where available), structure and reference for compounds in the public domain. +For n=2 FP data both individual values shown in parentheses. All SPR data n=1. ss, steady state.

Supplementary Table S2. Binding parameters determined for all compounds against mouse galectin-1 in FP and SPR assays.

Compound ID	Structure	Compound Series	FP Mean K_D (μM) ± SD^{+} (n)	SPR ss K_D (μM)	SPR kinetic K_D (μM)	SPR *kon* ($x10^{6}$ 1/Ms)	SPR *koff* (1/s)
**1* (GB0139 [11])**	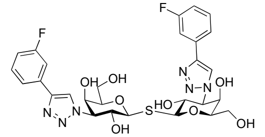	Disaccharide	0.116 ± 0.031 (3)	0.131	0.103	3.12	0.321
**2* (GB1107 [18])**	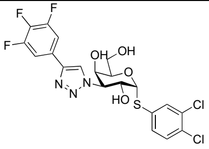	Monosaccharide	3.90 (1)	17.7	19.6	0.01	0.237
**3* (GB1211 [12])**	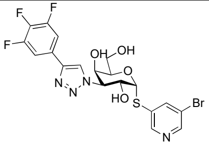	Monosaccharide	5.60 (1)	5.10	6.79	0.04	0.250
**4* (GB1490 [19])**	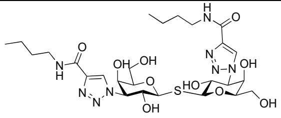	Monosaccharide	0.234 ± 0.025 (>3)	0.363	0.613	0.61	0.375
**5**	NA	Disaccharide	0.267 ± 0.087 (3)	0.409	0.417	0.53	0.222
**6**	NA	Monosaccharide	0.042 (1)	0.175	0.124	1.64	0.203
**7**	NA	Monosaccharide	0.038 ± 0.004 (3)	0.032	0.023	3.75	0.087

**8**	NA	Monosaccharide	0.063 (1)	0.117	0.132	2.78	0.366
**9**	NA	Monosaccharide	0.099 (0.091, 0.106)	0.171	0.185	1.56	0.288
**10**	NA	Monosaccharide	0.010 (1)	0.022	0.013	10.30	0.135
**11**	NA	Disaccharide	0.823 ± 0.170 (3)	1.04	0.964	1.74	1.68
**12**	NA	Disaccharide	0.317 ± 0.100 (3)	0.315	0.256	2.80	0.716
**13**	NA	Monosaccharide	0.072 ± 0.0087 (3)	0.164	0.092	2.38	0.220
**14**	NA	Disaccharide	8.30 (1)	5.13	7.64	0.42	3.18
**15**	NA	Monosaccharide	33.0 (1)	0.042	0.048	1.76	0.085

*Literature name (where available), structure and reference for compounds in the public domain. [+]For n=2 FP data both individual values shown in parentheses. All SPR data n=1. ss, steady state.

International Journal of *Molecular Sciences*

MDPI

Supplementary Table S3. The affinity differences between assay formats and species.

	**Fold K_D Differences**			
**Compound ID**	**Human Gal-1 FP *vs.* SPR**	**Mouse Gal-1 FP *vs.* SPR**	**FP Human *vs.* Mouse Gal-1**	**SPR Human *vs.* Mouse Gal-1**
**1* (GB0139 [11])**	0.9	1.1	9.7	1.3
**2* (GB1107 [18])**	1.9	4.5	1.1	2.5
**3* (GB1211 [12])**	1.2	0.9	1.8	1.4
**4* (GB1490 [19])**	1.8	1.6	0.6	0.6
**5**	3.6	1.5	2.4	1.0
**6**	8.3	4.2	2.2	1.1
**7**	0.9	0.8	2.3	2.1
**8**	2.3	1.9	0.8	3.0
**9**	1.8	1.7	0.9	2.2
**10**	2.1	2.2	1.2	1.8
**11**	1.8	1.3	1.5	1.2
**12**	58.2	1.0	4.5	12.9
**13**	1.6	2.3	1.3	1.8
**14**	0.9	0.6	1.0	0.7
**15**	1.8	0.6	2.1	0.6
**MEAN**	**5.9**	**1.7**	**2.2**	**2.3**

*Literature name and reference for compounds in the public domain. Affinity fold differences calculated by dividing the largest K_D by the smallest between assay and species comparisons.

References

11. Delaine, T.; Collins, P.; MacKinnon, A.; Sharma, G.; Stegmayr, J.; Rajput, V.K.; Mandal, S.; Cumpstey, I.; Larumbe, A.; Salameh, B.A.; et al. Galectin-3-Binding Glycomimetics that Strongly Reduce Bleomycin-Induced Lung Fibrosis and Modulate Intracellular Glycan Recognition. *Chembiochem* **2016**, *17*, 1759–1770. https://doi.org/10.1002/cbic.201600285.

12. Zetterberg, F.R.; MacKinnon, A.; Brimert, T.; Gravelle, L.; Johnsson, R.E.; Kahl-Knutson, B.; Leffler, H.; Nilsson, U.J.; Pedersen, A.; Peterson, K.; et al. Discovery and Optimization of the First Highly Effective and Orally Available Galectin 3 Inhibitors for Treatment of Fibrotic Disease. J. Med. Chem. 2022, 65, 12626–12638. https://doi.org/10.1021/acs.jmedchem.2c00660.

18. L. Vuong, E. Kouverianou, C.M. Rooney, B.J. McHugh, S.E. Howie, C.D. Gregory, S.J. Forbes, N.C. Henderson, F.R. Zetterberg, U.J. Nilsson, H. Leffler, P. Ford, A. Pederson, L. Gravelle, S. Tantawi, H. Schambye, T. Sethi, A.C. Mackinnon, An orally active galectin-3 antagonist inhibits lung adenocarcinoma growth and augments response to PD-L1 blockade, Cancer Res. 79 (2019) canres.2244.2018. https://doi.org/10.1158/0008-5472.can-18-2244.

19. Salameh, I. Cumpstey, A. Sundin, L.H. medicinal …, 1H-1,2,3-Triazol-1-yl thiodigalactoside derivatives as high affinity galectin-3 inhibitors, Bioorganic & Medicinal Chemistry. 18 (2010) 5367–5378. https://doi.org/10.1016/j.bmc.2010.05.040.
